# 

**Published:** 1973-01

**Authors:** 


The
Bristol Medico-Chirurgical Journal
(Incorporating the Medical Journal of the South-West)
Established 1883

Vol. 88 (i) JANUARY 1973 No. 325

### BRISTOL MEDICO-CHIRURGICAL JOURNAL

Editor: N. J. Brown, M.B., F.R.C.P., F.R.C.Path.

ublished Quarterly

Annual Subscription £2 Post Free

### BRISTOL MEDICO-CHIRURGICAL SOCIETY

**President 1972-73: Dr. James Macrae**

**President-elect: Dr. R. F. Barbour**

**Honorary Secretary: Dr. I. S. Bailey, 7 Percival Road, Bristol 8.**

**Honorary Treasurer: Dr. Frank Ross, Bristol Royal Infirmary, Bristol 2.**

The Society was founded in 1874. In 1883 publication of the Journal began and has continued quarterly ever since then. During the years 1949 to 1962 it appeared under the title of "The Medical Journal of the South West".

### THE BRISTOL MEDICO-CHIRURGICAL JOURNAL

**Editorial Committee**

| | |
|---|---|
| Editor | Dr. N. J. Brown, Southmead Hospital, Bristol |
| Assistant Editor | Dr. D. S. Reeves. |
| Members | Dr. Rhys Davies.<br>Dr. M. B. Lennard.<br>Professor R. C. Wofinden.<br>Dr. D. W. Wright.<br>The Hon. Treasurer and Hon. Secretary of the Society. |
| Business Manager | Dr. P. J. F. Baskett, Frenchay Hospital, Bristol. |
| Secretary | Mr. B. P. Jones, The Library, Medical School, University Walk, Bristol BS8 1TD. |

### NOTICE TO CONTRIBUTORS

The Journal is especially concerned to provide a place for the recording of medical thought, interests, and practice in and around Bristol. It therefore welcomes articles that, as well as being of immediate interest to members, will document the local and contemporary medical scene for our successors.

Original articles are invited provided that they have not been published elsewhere. References in the text should be by author's name, with year of publication in parenthesis; they should be listed at the end in alphabetical order, the name of each journal or book being given in full—not abbreviated—and the title of the article added. Illustrations should be supplied ready for reproduction. Line drawing should be in Indian ink on firm white paper or board. The author will be informed if it is found necessary to ask him to pay for the making of blocks.

The following contributions will be especially welcome; notes of forthcoming events, meetings held, degrees conferred, staff appointments, honours and public appointments and other local news.

### The Medical Library

In 1890 the Bristol Medico-Chirurgical Society founded a medical library which in 1892 was combined with that of the Bristol Medical School. This became the University of Bristol Medical Library in 1925 and an agreement in that year confirmed the privilege of Members of the Society to use the University Medical Library.

### A SELECTION OF RECENT ADDITIONS TO THE MEDICAL LIBRARY

MARRABLE, A. W.: The embryonic pig. (Pitman)

MITCHELL, A. R. K.: Psychological medicine in family practice. (Baillière)

MULLIGAN, H. W., ed.: The African trypanosomiases. (Allen & Unwin). Presented by the Association of Applied Biologists.

MURRAY, M. R. & KOPECH, G.: A bibliography of the research in tissue culture 1884 to 1950. (Acad. Press)

ORAM, S.: Clinical heart disease. (Heinemann)

PÉRON, F. G. & CALDWELL, B. V., eds.: Immunologic methods in steroid determination. (Appleton-Century-Crofts). Presented by the Association of Applied Biologists.

POLLI, E., ed.: Neurochemistry of hepatic coma. (Karger). Presented by the Association of Applied Biologists.

PRACY, R. et al.: A short textbook of ear, nose and throat. (English Univ. Press)

PRYOR, W. J. & MACALISTER, A. D.: General anaesthetic and sedation techniques for dentistry. (Wright). Presented by John Wright & Sons Ltd.

RAMSEY, P.: The patient as person: explorations in medical ethics. (Yale Univ. Press)

RECHIGL, M. ed.: Enzyme synthesis and degradation in mammalian systems. (Karger)

REITH, E. J. & ROSS, M. H.: Atlas of descriptive histology. Ed 2. (Harper & Row)

RENDLE-SHORT, J.: The child: a textbook for the paediatric team. (Wright). Presented by John Wright and Sons Ltd.

RIBBONS, D. W. et al., eds.: Nucleic acid-protein interactions and nucleic acid synthesis in viral infection. (North-Holland)

ROBERTS, T.: A handbook for psychiatric nurses. (Wright). Presented by John Wright & Sons Ltd.

ROBINSON, D.: The process of becoming ill. (Routledge & Kegan Paul)

ROBISON, G. A. et al.: Cyclic AMP. (Acad. Press)

ROGERS, A. F. & SUTHERLAND, R. J.: Antarctic climate, clothing and acclimatization: final scientific report. (E.O.A.R. 69-0068) (Bristol Univ. Dept. of Physiology)

RUBIN, F.: Learning and sleep: the theory and practice of hypnopaedia. (Wright). Presented by John Wright and Sons Ltd.

SCHAMROTH, L.: Disorders of cardiac rhythm. (Blackwell)

SCHETTLER, G. ed.: Platelets and the vessel wall fibrin deposition. (Thieme)

SCHUMANN, H. J. & KRONEBERG, G. eds.: New aspects of storage and release mechanisms of catecholamines. (Springer)

SHARRARD, W. J. W.: Paediatric orthopaedics and fractures. (Blackwell)

SILVESTRI, L. G. ed.: Biology of oncogenic viruses. (North-Holland)

SMITH, P. F.: Biology of mycoplasmas. (Acad. Press)

STEINER, M. M.: Clinical approach to endocrine problems in children. (Mosby)

STEWART, G. T. & McGOVERN, J. P. eds.: Penicillin allergy. (Thomas)

STORZ, J.: Chlamydia and chlamydia-induced diseases. (Thomas)

SUSSER, M. W. & WATSON, W.: Sociology in medicine, Ed 2. (Oxford Univ. Press)

TAUSK, M. ed.: Pharmacology of the endocrine system and related drugs: progestational drugs and antifertility agents. (Pergamon)

VAETH, J. M. ed.: The interrelationship of chemotherapeutic agents and radiation therapy in the treatment of cancer (Karger)

VAETH, J. M. ed.: Interrelationship of surgery and radiation therapy in the treatment of cancer. (Karger)

VOGEL, F. & RÖHRBORN, G. eds.: Chemical mutagenesis in mammals and man. (Springer)

WAKIL, S. J. ed.: Lipid metabolism. (Acad. Press)

WERSHUB, L. P.: Urology: from antiquity to the 20th century. (Warren H. Green)

WHEELER, R. C.: An atlas of tooth form. Ed 4. (Saunders)

WHEELER, R. C.: A textbook of dental anatomy and physiology. Ed 4. (Saunders)

WILLIAMS, C. B.: Basic practical surgery. (Wright). Presented by John Wright & Sons Ltd.

WILLS, M. R.: Biochemical consequences of chronic renal failure. (Harvey Miller & Metcalf)

WOODLIFF, H. J.: Leukaemia cytogenetics. (Lloyd-Luke)

WRIGHT, M. I.: The pathology of deafness: an introduction. (Manchester Univ. Press)

ZORAB, P. A. ed.: Scoliosis and growth. (Churchill Livingstone)

ZUBEK, J. P. ed.: Sensory deprivation: fifteen years of research. (Appleton-Century-Crofts)

